# Sex differences in incidence rate, and temporal changes in surgical management and adverse events after hip fracture surgery in Denmark 1997–2017: a register-based study of 153,058 hip fracture patients

**DOI:** 10.1080/17453674.2021.1923256

**Published:** 2021-05-14

**Authors:** Liv R Wahlsten, Henrik Palm, Gunnar H Gislason, Stig Brorson

**Affiliations:** a Department of Orthopaedics, Copenhagen University Hospital Herlev-Gentofte; b Department of Orthopaedics, Copenhagen University Hospital Bispebjerg; c Department of Cardiology, Research 1, Copenhagen University Hospital Herlev-Gentofte; d Department of Orthopaedic Surgery, Zealand University Hospital Køge, Denmark

## Abstract

Background and purpose — Extensive research and national multidisciplinary programs have striven to introduce uniform standards of treatment and mitigate mortality and adverse events after hip fracture surgery over the past decades. A large-scale overview of temporal developments in hip fracture surgery and care is warranted.

Patients and methods — We studied Danish patients aged ≥ 60 years, sustaining their first ever hip fracture between 1997 and 2017. Patients were identified from the Danish National Patient Registry (DNPR). Incidence rates of first hip fracture were calculated per 1,000 patient-years and stratified by age group and sex. Information on pre-injury living settings, comorbidities, and medications were obtained from national administrative registers. Type of fracture and treatment choice were recorded, and patients were followed for 1 year to observe mortality, readmission, and surgical complications.

Results — Data from 153,058 patients was analyzed. Incidence rate decreased in both sexes, but only led to a reduction in the annual number of hip fractures in the female population. Choice of surgery shifted away from sliding hip screws and parallel implants (SHS-PI), towards intramedullary nailing and hemi-/arthroplasties for trochanteric and femoral neck fractures, respectively. Pre-injury diagnosed morbidity and 1-year readmissions increased contrary to mortality. Median age remained stable around 83 (IQR 77–88) for women and 80 (IQR 73–86) for men.

Interpretation — Over the past 2 decades important aspects of hip fracture management have improved. However, sex differences were observed, and men remain more vulnerable than women in terms of morbidity, mortality, and incidence rate.

The collateral effects of hip fractures are tremendous in terms of healthcare costs, morbidity, mortality, and lost quality of life for the patients and their families (Vochteloo et al. 2013, Schemitsch et al. 2019). Increasing incidence of hip fractures in the last 50 years, longer life expectancy, and large birth cohorts during World War II have predicted a significant rise in the incidence of hip fractures in the coming decades (Cooper et al. 1992, 2011, Rosengren and Karlsson 2014).

In the past 20 years, extensive research has striven to develop and amend fracture prevention, algorithms for operative treatment, and multidisciplinary approaches to care and rehabilitation, in order to improve survival and mitigate adverse events after hip fractures. Many aspects thereof have been analyzed, changed, and evaluated as the perception of hip fracture surgery has shifted from routine surgery often performed by younger surgeons to a global ambition for complex highly specialized treatment regimens in multidisciplinary settings. In 1999 the first national Danish reference program for the management of hip fractures emerged as a part of a government-initiated National Indicator Project (NIP) aimed to improve uniformity and quality. The aim was to suggest, describe and implement quality indicators suitable for benchmarking and monitoring the management of hip fractures in Denmark across regions and departments. Since then, several national cross-sectional quality projects have been deployed.

A large-scale overview of temporal developments in hip fracture surgery and care is warranted to help clinicians, politicians, and caretakers to see new opportunities to improve safety and care for patients with hip fractures in the future.

In this study we report how the Danish hip fracture population and management have changed over 2 decades in terms of incidence rates, pre-fracture comorbidity, choice of primary implant, surgical complications, readmissions, and mortality after hip fracture surgery.

## Patients and methods

Throughout the study period, the entire Danish population of 5.6 million inhabitants has been covered by tax-financed public health and social care insurance, securing free and equal access to healthcare services free of personal charge.

### Study population

All Danes aged ≥ 60 years undergoing their first operative procedure due to a hip fracture, between 1997 and 2017, were identified and followed up 1 year from the day of admission or until death, whichever came first. Patients were included if they had a hip fracture diagnosed and surgery in the same hospital stay or overlapping hospitals stays. Patients with prior diagnosis or procedures compatible with having a hip fracture were excluded. The positive predictive value of the hip fracture diagnosis code and hip fracture surgical procedure codes in DNPR are above 90% separately (Hjelholt et al. 2020). By combining diagnosis and surgery codes, the positive predictive value of the hip fracture diagnoses was suspected to be even higher. ICD-8 and 10 codes and NSCP codes used to define the population are listed in Table 1 (see Supplementary data).

**Table 1.** Codes used to define the population

Fracture diagnosis	ICD-10 codes	ICD-8
Femoral neck fracture	S72.0	820
Trochanteric fracture	S72.1 + 72.2	820
Other diagnosis		
Atrial fibrillation	I48	
Cancer **^a^**	C00-42, C44-96	140-209
COPD	J40-J47	490-492
Dementia	G30, F00-F03	290
Depression	F32-F33	2969 + 2969 + 2980 + 3004
Diabetes	E10-14	249 + 250
Heart disease	I00-09, I20-99	410-414 + 420-429
Osteoporosis	M80-85	342
Parkinsons disease	G20	723
Procedures	SKS/NSCP codes ^b^	
PI-SHS	KNFJ:30-33 + 40-43 + 60-63 + 70-73 + 90 + 93	
IMN	KNFJ:50-53 + 80-83	
Hemi-/arthroplasty	KNFB:02 + 03 + 09 + 12 + 13 + 20 + 30 + 40 + 99	
Dislocation of arthroplasty	KNFH	
Removal of implant	KNFU	
Infection	KNFW59 + 69	
Medication	ATC-codes	
Anti-dementia therapy	N06D	
Anti-depressants	N06A	
Anti-parkinson medication	N04	
Glucose lowering drugs	A10	

**^a^**Cancers of the skin is not included

**^b^** SKS is the Danish version of the NCSP system.

### Data sources

In the Danish Central Population Register a unique and permanent civil registration number for each individual resident in Denmark is held. Upon contact with any part of the public system, e.g., healthcare services, the civil registration number is registered in the appropriate administrative register along with an action code. Linkage of information across registries is feasible on the individual level through the civil registration number.

Registrations from 5 different national administrative registries were retrieved in this study: the Central Population Registry, Danish National Patient Registry, National Prescription Registry, Nursing Home Registry, and Household Income Registry.

### Pre-injury status, type of fracture, and choice of implants

Information on pre-existing comorbidity, defined by the international ICD-10/8 system, was retrieved from the Danish National Patient Registry (DNPR). Diagnoses given or renewed 10 years prior to admission were considered active. To ensure accuracy some conditions, e.g., chronic obstructive pulmonary disease (COPD), diabetes, and depression, were defined from a combination of diagnosis and drug usage. Data on concomitant medications according to Anatomical Therapeutic Chemical Classification (ATC) codes was collected from the National Prescription Registry. Claimed prescriptions within 6 months prior to admission for hip fracture were considered to represent active treatment. Information on pre-injury living settings, i.e., living alone or co-living, was drawn from the Household Income Registers provided by Statistics Denmark based on “family type.” Living in a nursing home prior to admission was defined as the shift from living in an unstaffed private home to a permanent institutional residence. Based on procedure codes registered in the DNPR, surgical treatment was categorized as either “arthroplasty”—covering hemi- and total arthroplasties, intra-medullary nailing (IMN), or sliding hip screws (SHS) and parallel implants (PI), collectively referred to as SHS-PI. SHS and PI were gathered into one category because of overlapping indications and the treatment choice is mostly determined by local tradition. Data on surgical complication rates was retrieved from the DNPR and defined as the 1st occurrence of removal of an implant, reoperation due to superficial or deep infection or dislocation of arthroplasty, within 1 year of surgery

### Statistics

Differences in the distribution of comorbidity and living setting at baseline, between males and females, were calculated using Student’s t-test for continuous variables and a chi-square test for categorical variables. Age and sex stratified incidence rates were calculated in a time-updated model as number of first ever hip fractures per 1,000 person-years in that age and sex category, where the number of person years and new events, in each category, were updated each month. Cumulative incidence functions were applied when time to event and absolute crude risks were of interest, i.e., mortality and readmissions. For readmissions, the Aalen–Johansen estimator was used in amendment of the cumulative incidence function to calculate competing risk of death. 95% confidence intervals (CI) were calculated for all estimated parameters. All analyses were performed with SAS statistical software version 9.4 (SAS Institute Inc, Cary, NC, USA) and R-studio version 3.2.2 (2016-10-31) (R Foundation for Statistical Computing, Vienna, Austria).

### Ethics, funding, and potential conflicts of interest

In Denmark, registry-based studies do not require ethical committee approval or patient consent if the study is conducted for the sole purpose of statistics and scientific research as defined in the Data Protection Act. Approval to use the data sources for this research project was granted by the data responsible institute in the Capital Region of Denmark in accordance with the General Data Protection Regulation (approval number, P-2019-404). This research did not receive grants from any funding agency in the public, commercial, or not-for-profit sectors. There are no conflicts of interest to declare for any of the authors.

## Results

### Incidence of hip fractures

**Figure 1.**
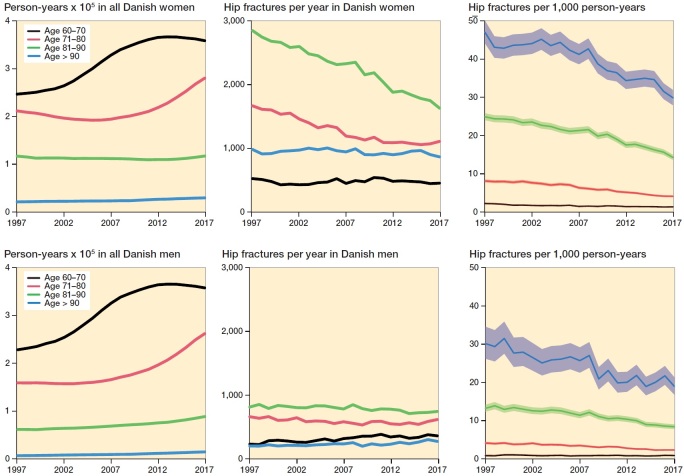
Incidence of hip fracture in the Danish population 1997–2017. a. Top row. Person-years in the entire Danish population of women, absolute number of hip fractures per year among women, and incidence rate of hip fractures among women, all displayed in age strata. b. Bottom row. Person-years in the entire Danish population of men, absolute number of hip fractures per year among men, and incidence rate of hip fractures among men, all displayed in age strata.

From 1997 to 2017, 153,058 Danish patients sustained their first hip fracture. The annual number of first hip fractures fell gradually during the entire study period from 8,127 patients in 1997 to 6,110 in 2017 (Table 2). In Figure 1a incidence rates of patients suffering their first hip fracture stratified by age and sex are displayed. Absolute crude annual numbers of first hip fractures and person-years in the Danish population are also displayed to demonstrate the demographic development in each age stratum. A steady decrease in incidence was seen in all age groups in the female hip fracture population. The decrease in incidence rate among the 71–80-year-old women from 7.9 (CI 7.5–8.2) per 1,000 person years (kPY) to 3.9 (CI 3.7–4.2) per kPY and the 81–90-year-old women from 24 (CI 23–25) per kPY to 14 (CI 13–15) per kPY (Figure 1a) had the largest impact on the total annual number of first hip fractures. The curve flattened in the last part of the study period for the 71–80-year-olds, due to large birth cohorts during World War II. A more modest reduction in incidence was observed in men (Figure 1b). The maximum decrease was seen among the 71–80-year-olds where a reduction from 4.2 (CI 3.9–4.5) to 2.4 (CI 2.2–2.6) per kPY led to a slight fall in annual number of hip fractures up to 2012 but was exceeded by the rise in person-years from 2013 onwards. Overall, the increase in person-years introduced by the large birth cohorts during World War II and increasing life expectancy was balanced by a decrease in incidence in men but exceeded by the decrease in women, which led to a stable annual number of fractures in men and a decrease in women.

**Table 2.** Study population with living status, fracture type, and pre-existing comorbidity. Values are count (%) unless otherwise specified

	1997			2007			2017		
	Female	Male		Female	Male		Female	Male	
Factor	n = 6,144	n = 1,983	p-value	n = 5,016	n = 1,989	p-value	n = 4,079	n = 2,031	p-value
Age (IQR)	83 (77–88)	80 (74–86)	< 0.001	83 (77–88)	81 (73–86)	< 0.001	83 (76–89)	80(72–87)	< 0.001
Living alone	4,541 (74)	996 (50)	< 0.001	3,568 (71)	1,017 (51)	< 0.001	3,090 (76)	1,171 (58)	< 0.001
Resident in nursinghome	1,136 (19)	306 (15)	0.002	982 (20)	333 (17)	0.007	429 (11)	179 (8.8)	0.04
Femoral neck fractures	3,422 (56)	1,158 (58)	0.04	2,690 (54)	1,030 (52)	0.2	2,213 (54)	1,097 (54)	0.9
Diabetes	449 (7.3)	183 (9.2)	0.006	569 (11)	303 (15)	< 0.001	551 (14)	364 (18)	< 0.001
Depression	1,410 (23)	317 (16)	< 0.001	1,707 (34)	583 (29)	< 0.001	1,163 (29)	462 (23)	< 0.001
Parkinsons disease	277 (4.5)	115 (5.8)	0.02	173 (3.4)	111 (5.6)	< 0.001	169 (4.1)	125 (6.2)	0.001
Dementia	354 (5.8)	107 (5.4)	0.6	751 (15)	293 (15)	0.8	610 (15)	261 (13)	0.03
Heart disease	1,789 (29)	700 (35)	< 0.001	2,293 (46)	1,112 (56)	< 0.001	1,975 (48)	1,182 (58)	< 0 .001
COPD ^a^	326 (5.3)	206 (10)	< 0.001	539 (11)	259 (13)	0.008	494 (12)	288 (14)	0.03
Osteoporosis	464 (7.6)	33 (1.7)	< 0.001	810 (16)	160 (8.0)	< 0.001	1,101 (27)	261 (13)	< 0.001
Cancer	650 (11)	263 (13)	0.001	648 (13)	364 (18)	< 0.001	687 (17)	438 (22)	< 0.001

**^a^** Chronic obstructive pulmonary disease.

### Pre-injury status

On average women were 2.6 years older than men when sustaining their first hip fracture, with a median age of 83 years (interquartile range [IQR] 77–89). Only small variations were seen in age over time. More women were living alone. The proportion of patients who were resident in nursing homes at the time of injury was higher in 1997 and fell successively in 2007 and 2017 for both men and women (Table 2). The burden of diagnosed diseases before sustaining a hip fracture increased during the study period for both sexes, but in general men had more diagnosed diseases than women except for depression and osteoporosis, which were more common in women (Table 2).

### Type of fracture, choice of treatment, and surgical complications

**Figure 2.**
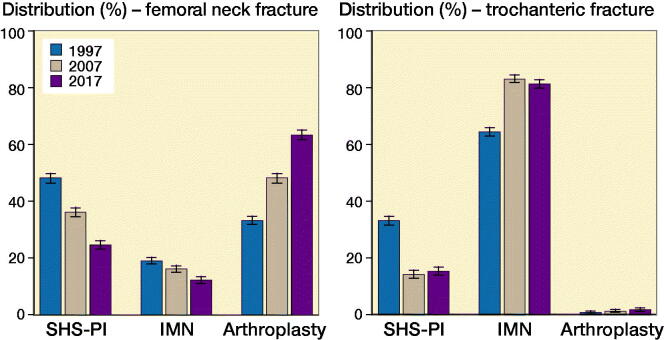
Temporal trends in operative management of hip fractures. SHS-PI: Sliding hip screws and parallel implants, IMN: Intramedullary nailing.

**Figure 3.**
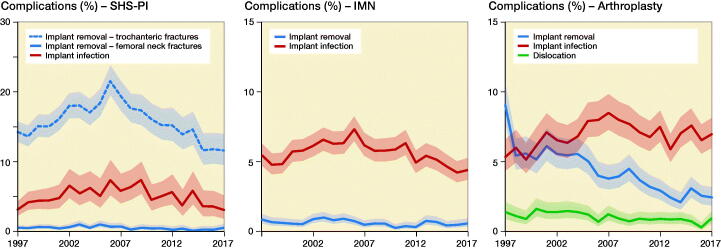
Surgical complications in the first postoperative year. SHS-PI: Sliding hip screws and parallel implants, IMN: Intramedullary nailing.

In Figure 2, the choice of surgical treatment according to fracture type is shown for the years 1997, 2007, and 2017. The use of SHS-PI fell gradually throughout the study period from 48% (CI 47–49) and 34% (CI 32–35) in femoral neck and trochanteric fractures in 1997 to 25% (CI 23–26) and 16% (CI 15–17) in 2017, respectively, and were replaced by an increase in arthroplasties and intramedullary nailing. The proportion of trochanteric versus femoral neck fracture was stable. Fracture-related complications or secondary surgery measured by removal of implants, implant related infections, or dislocations of arthroplasties in the first postoperative year is shown in Figure 3. Removal of implants was more frequent after SHS-PI of femoral neck fractures, compared with SHS-PI of trochanteric fractures and intramedullary nailing and arthroplasties. The latter showed a decrease in implant removal over time, but also a tendency towards an increase in dislocations.

### Length of stay, readmission, and mortality

**Figure 4.**
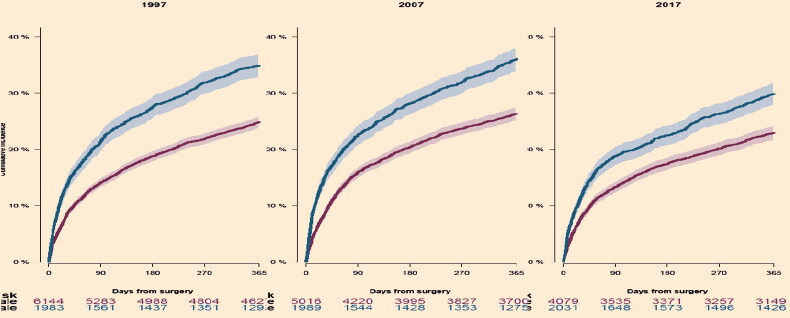
All-cause readmission after hip fracture surgery.

The median length of stay in hospital shortened from 13 days (IQR 7–22) in 1997 to 6 days (IQR 4–9) in 2017 for both sexes (Table 3). There was an inverse relationship between absolute risk of readmission and time from discharge, which can be observed in the slope of the cumulative incidence curve in Figure 3. The rate and pace of readmission were similar in 2007 and 2017, but lower in 1997 (Figure 4).

**Table 3.** Median length of stay in hospital and cumulative postoperative mortality. Values are count (%) unless otherwise specified

	1997			2007			2017		
	Female	Male		Female	Male		Female	Male	
Factor	n = 6,144	n = 1,983	p-value	n = 5.016	n = 1,989	p-value	n = 4,079	n = 2,031	p-value
Length of stay (IQR)	13 (7–22)	13 (7–21)	NA	9 (5–13)	9 (5–13)	NA	6 (4–9)	6 (4–9)	NA
In hospital mortality	324 (5.3)	167 (8.4)	< 0.001	165 (3.3)	130 (6.5)	< 0.001	100 (2.5)	102 (5.0)	< 0.001
30-day mortality	501 (8.2)	266 (13)	< 0.001	413 (8.2)	291 (15)	< 0.001	316 (7.7)	235 (12)	< 0.001
1-year mortality	1,568 (25)	711 (36)	< 0.001	1,335 (27)	724 (36)	< 0.001	942 (23)	612 (30)	< 0.001

**Figure 5.**
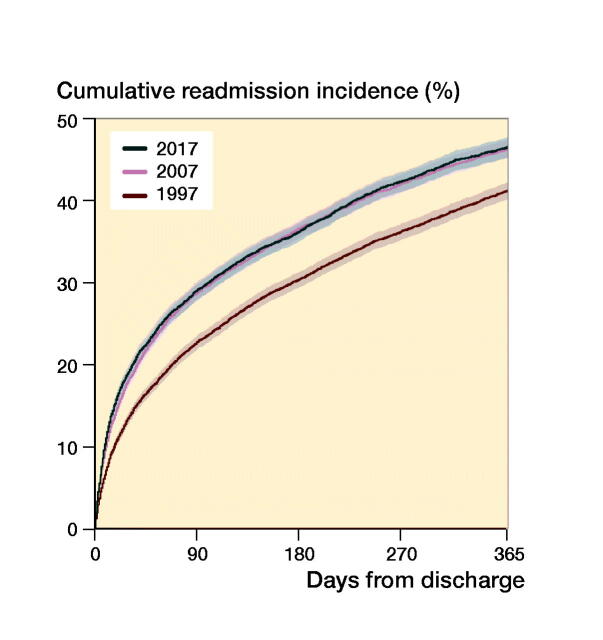
1-year mortality after hip fracture surgery.

The 1-year cumulative incidence of postoperative mortality was similar in 1997 and 2007 but fell gradually from 2007 to 2017, to 30% and 23% for men and women (Table 3, Figure 5). Mortality was higher among men at all measured timepoints.

## Discussion

### Key findings

In this study of 153,058 Danish patients who suffered their first hip fracture in 1997–2017, we found the incidence and the overall annual number of hip fractures to be steadily declining, especially for women, despite strong secular trends towards an ageing population. During the study period the average age of primary hip fracture was unchanged, while the level of pre-injury comorbidities increased. For both femoral neck and trochanteric fractures, the treatment preference moved away from SHS-PI towards arthroplasties and intramedullary nailing, respectively. The median length of stay in hospital shortened contrary to readmission rates, which were lower in 1997 compared with 2007 and 2017. Complications showed noticeable fluctuations over time. Mortality decreased in all measures for both sexes in the last decade of observation compared with the first.

### Incidence and comorbidities of hip fractures

The incidence of hip fractures increased in many parts of the world until the late 1990s; around the millennium a plateau effect was described and after that a fall in incidence has been observed in many industrialized countries (Ahlborg et al. 2010, Sullivan et al. 2016, Rosengren et al. 2017). It has been debated whether the decrease in incidence rate of hip fractures would be enough to balance the effect of a growing population of elderly individuals (Korhonen et al. 2013, Rosengren and Karlsson 2014, Lewiecki et al. 2018). In the female population the decrease in incidence was sufficient to cause fewer annual hip fractures, whereas the incidence rate in the male population only kept the total annual number at a stable level. The cause of the difference between the sexes is unexplained, but merits further research as it may hold a causal inference that can reveal modifiable factors which can aid prevention in the future. One important aspect is the difference in prevalence of osteoporosis between women and men, where women have a 2–3 times higher prevalence than men (Abrahamsen and Vestergaard 2010) and men may exhibit different disease patterns than women (Haentjens et al. 2004). The extensive focus on osteoporosis in women may have led to a perception of osteoporosis as a women’s disease, leading to a negative detection bias and more pronounced diagnostic and treatment deficits in men (Vestergaard et al. 2005).

Pre-existing comorbidity is a strong predictor of rehabilitation outcome and mortality after hip fracture (Roche et al. 2005, Vestergaard et al. 2007, Sheehan et al. 2018). From 1997 to 2017 a substantial increase in the proportion of patients with comorbidity was observed for several diagnoses i.e., diabetes, osteoporosis, COPD, cancer, heart disease, and dementia. Rather than an expression of declining health status in the hip fracture population, this development represents the increased focus on health and wellbeing of the elderly over the past 20 years, leading to more rigorous examinations, better diagnosis, and more treatment deployed earlier in the course of a disease. This aspect correlates well with improved survival in the hip fracture population and the rise in mean life expectancy in the general population.

### Type of fracture, choice of treatment, and complications

Operative management of hip fractures has been a topic of debate for many years, regarding choice of procedure, implant, approach, and anesthetic strategy. In the last 20 years evidence-based treatment algorithms have been introduced and widely used to determine surgical treatment in Denmark (Palm et al. 2012). Focus on uniform treatment guidelines may have contributed to the shift from SHS-PI toward arthroplasties and intramedullary nailing for femoral neck and trochanteric fractures, respectively (Rogmark and Leonardsson 2016). Complications leading to removal of implants have fluctuated in frequency over the years, especially for patients with femoral neck fractures who were treated with SHS-PI. The term “complication” is widely used for secondary procedures, but may be inappropriate in some cases, especially for removal of implants. While the majority of implant removals after femoral neck fractures are likely to emanate from femoral head necrosis, cut-out of implants, and non-union problems, some implant removals may be requested by the patient due to minor soft tissue irritation after successful bone healing. Detailed information regarding the cause of removal was not obtainable for this study. For SHS-PI and intramedullary nailing, there was a tendency to a rise in frequency from 1997 to 2007, but a decline from 2007 onwards, leading to an overall reduction during the study period. Local initiatives to reduce complication rates may show clear efficacy that is not detectable on a national level.

In 2 prospective Swedish cohort studies of 1,111 hip fracture arthroplasties, dislocations were 3–4 times higher with the traditional posterolateral approach compared with an anterolateral approach (Enocson et al. 2008, Sköldenberg et al. 2010), while the only RCT between the lateral approach or the piriformis-saving posterior approach used for 216 patients by the same primary surgeon saw no difference (Parker 2015). In Denmark the posterolateral approach has been widely used for decades despite the unchanged high dislocation rate. Increasing focus has, however, emerged in the most recent years with 2 schools of thought: either preserving the piriformis tendon or implementing the anterolateral approach.

### Length of stay, readmission, and mortality

The introduction of fast-track programs for hip fracture patients in the late 1990s has been widespread, although only partially implemented in many centers (Egerod et al. 2010). The benefits of short total length of stay are controversial, but most studies find that this does not appear to be harmful (Haugan et al. 2017, Pollmann et al. 2019) down to a certain level. As in other countries, the median length of hospital stay was reduced in Denmark from 1997 to 2017, but an increase in frequency and pace of readmission was observed collaterally (Figure 4).

The rise in frequency of readmission could be multifactorial, e.g., changes in the standard of care, enhanced survival that made more patients eligible for readmission, changes in discharge criteria leading to premature discharge, or a lower threshold for hospital admissions in general etc. This area deserves further investigations.

In the first 10 years, a reduction in mortality was seen in in-hospital mortality. During the second 10 years of observation 30-day mortality and 1-year mortality declined as well. Over time a decrease in mortality was seen for both sexes, but men had higher mortality at all measured timepoints throughout the study period, compared with women (Figure 5 and Table 2), which is in accordance with other hip fracture populations and study methods (Kannegaard et al. 2010, von Friesendorff et al. 2016, Pollmann et al. 2019). The gradual improvement in mortality complies well with the overall hip fracture efforts in Denmark, which initially focused on the in-hospital management of hip fractures, and during the past century expanded to include after-care and community-based rehabilitation. An important derived effect of the awareness that high-quality care for hip fracture patients comprises more than choosing the right implant is the implementation of orthogeriatric care. In a prospective cohort study from UK the association between mortality and implementation of orthogeriatric teams, based on 33,152 hip fracture patients, showed hazard ratios of 0.73 (CI 0.62–0.82) and 0.81 (CI 0.75–0.87) for 30 day and 1-year mortality, respectively (Hawley et al. 2016).

### Strength and limitations

This study offers an overview of developments and time trends in a broad spectrum of aspects in hip fracture surgery. Similar studies are difficult to undertake in most other countries.

The major strength of this study is the large number of patients free of selection, surveillance, and recall biases. Also, the high reliability of the Danish administrative register system, where registrations are mandatory for departmental reimbursement of health and social care expenses, ensures the trustworthiness of these results. It is reasonable to assume that these results transfer to other populations than the Danish, especially to populations with similar living standards, life expectancy, and access to healthcare. A major limitation in this study is the lack of clinical observations and context, which could allow more detailed analysis and causal interpretations. Other limitations are inherent to the nature of this study and may have led to conservative estimates, for instance only complications that lead to surgery/hospital contact were registered, thus only patients alive, capable, and eligible for secondary surgery appear in this count.

## Conclusion

Over the past 2 decades, a decrease in incidence rate and mortality has been accomplished for the hip fracture population. The median age of first hip fracture was stable throughout the study period despite increasing life expectancy and more comorbidities at the time of fracture. Men remain more vulnerable measured by postoperative mortality compared with women and showed a lower tendency to a decrease in incidence rate of hip fractures. Choice of surgery shifted away from SHS-PI, toward intramedullary nailing and hemi-/arthroplasties for trochanteric and femoral neck fractures, respectively. Further research is warranted to determine causality of the gender difference in incidence rate that we observed in this study as it may play an important role in preventive strategies in the future.

## Supplementary Material

Supplemental Material

## Supplementary data

Table 1 is available as supplementary data in the online version of this article, http://dx.doi.org/10.1080/17453674.2021.1923256
