# Supplementary material for: Sex differences in incidence rate, and temporal changes in surgical management and adverse events after hip fracture surgery in Denmark 1997–2017: a register-based study of 153,058 hip fracture patients
Source: Acta Orthop. 2021 May 14;92(4):424–30. doi: 10.1080/17453674.2021.1923256 (PMC8381898; doi:10.1080/17453674.2021.1923256)
Supplement: Supplemental Material [file IORT_A_1923256_SM6339.pdf]

Supplementary data

Table 1. Codes used to define the population

Fracture diagnosis	ICD-10 codes	ICD-8
Femoral neck fracture	S72.0	820
Trochanteric fracture	S72.1 + 72.2	820
Other diagnosis		
Atrial fibrillation	I48	
Cancer [a]	C00-42, C44-96	140-209
COPD	J40-J47	490-492
Dementia	G30, F00-F03	290
Depression	F32-F33	2969+2969+2980+3004
Diabetes	E10-14	249+250
Heart disease	I00-09, I20-99	410-414+ 420-429
Osteoporosis	M80-85	342
Parkinsons disease	G20	723
Procedures	SKS/NSCP codes [b]	
PI-SHS	KNFJ:30-33+40-43+60-63+70-73+90+93	
IMN	KNFJ:50-53+ 80-83	
Hemi-/arthroplasty	KNFB:02+03+09+12+13+20+30+40+99	
Dislocation of arthroplasty	KNFH	
Removal of implant	KNFU	
Infection	KNFW59+69	
Medication	ATC-codes	
Anti-dementia therapy	N06D	
Anti-depressants	N06A	
Anti-parkinson medication	N04	
Glucose lowering drugs	A10	

[a] Cancers of the skin is not included
[b] SKS is the Danish version of the NCSP system.
